# Genome-wide characterization and expression analysis of the B3 superfamily in response to abiotic stresses in alfalfa (*Medicago sativa* L.)

**DOI:** 10.1186/s12870-026-09277-0

**Published:** 2026-06-26

**Authors:** Yajing Wu, He Zhu, Siqi Wang, Yuxuan Ding, Tao Zhu, Qingchuan Yang, Xue Wang

**Affiliations:** Institute of Animal Science, Chinese Academy of Agricultural Sciences, Beijing, 100193 China

**Keywords:** Alfalfa, B3 superfamily, Transcription factor, Abiotic stress, Phosphorus

## Abstract

**Background:**

Alfalfa (*Medicago sativa* L.), a key perennial leguminous forage, suffers severe yield and quality losses under diverse abiotic stresses. The plant-specific B3 superfamily transcription factors regulate plant growth, development and abiotic stress responses. However, their systematic identification and stress-responsive expression patterns in alfalfa remain uncharacterized, especially under salt, drought, cold, and low-phosphorus stresses.

**Results:**

A total of 146 *MsB3* genes were identified from the genome of alfalfa cultivar Zhongmu No.1 and classified into four subfamilies with distinct member numbers, 89 belonging to the REM subfamily, 42 to the ARF subfamily, 8 to the LAV subfamily and 7 to the RAV subfamily. All *MsB3* genes were renamed based on their chromosomal distribution and subfamily classification. Gene structure and conserved motif analyses of *MsB3* genes showed variable exon counts, subfamily-specific motifs, and a conserved B3 DNA-binding domain in all MsB3 proteins. Collinearity analysis detected 18 tandem and 13 segmental duplication events. Transcriptomic data demonstrated tissue-specific expression for three *MsB3* genes, while 73 *MsB3* genes showed widespread expression across six alfalfa tissues including elongated stems, post-elongated stems, flowers, leaves, nodules and roots. Further analysis of transcriptomic data under salt, drought, cold and low-phosphorus stresses identified numerous stress-responsive *MsB3* members, with salt stress inducing the largest number of responsive genes at 25 and low-phosphorus stress eliciting the fewest at 12. Among these stress-responsive genes, 14 genes exhibited stress-specific expression patterns, seven responded to three different abiotic stresses, and only *MsARF16* was responsive to all four stresses, the expression pattern of which was further validated by qRT-PCR.

**Conclusion:**

This study systematically identified and characterized the B3 superfamily genes in alfalfa. Abiotic stress-responsive candidate genes were identified via transcriptomic analysis under multiple stress conditions. The findings furnish essential insights into subsequent functional investigations of alfalfa *B3* genes and the genetic improvement of alfalfa abiotic stress resistance.

**Supplementary Information:**

The online version contains supplementary material available at 10.1186/s12870-026-09277-0.

## Background

Alfalfa (*Medicago sativa* L.), a perennial legume forage known as the “queen of forages”, is widely cultivated worldwide for its high nutritional value, strong biological nitrogen fixation capacity, and wide adaptability [1–3]. However, the sustainable production of alfalfa is increasingly challenged by global climate change and soil resource constraints, with abiotic stresses becoming the major limiting factors for its yield and quality stability. Therefore, mining key stress-resistant regulatory genes and clarifying their molecular mechanisms have become pivotal targets for alfalfa stress-resistance molecular breeding [4]. The recent release of high-quality chromosome-level reference genomes of alfalfa has laid a solid foundation for genome-wide identification of functional gene families and dissection of stress regulatory networks [5–7].

Salt, drought, cold, and low-phosphorus stress are the four most critical abiotic constraints restricting alfalfa planting and production [8, 9]. Salt stress disrupts cellular Na^+^ and K^+^ homeostasis, triggers osmotic stress and ion toxicity, and ultimately inhibits alfalfa vegetative growth and forage yield [10]. Drought stress induces excessive reactive oxygen species accumulation and oxidative damage, suppresses photosynthetic efficiency, and restricts biomass accumulation in alfalfa [11, 12]. Cold stress not only impairs photosynthesis and cell membrane stability in alfalfa, but also alters its dormancy characteristics, directly reducing overwintering survival rate and spring regeneration capacity [13]. As an essential macronutrient for plant growth and development, phosphorus is particularly critical for alfalfa. As a leguminous forage, alfalfa relies heavily on phosphorus to sustain symbiotic nitrogen fixation, root system construction, and biomass synthesis. However, more than 30% of global arable land suffers from phosphorus deficiency, which severely inhibits alfalfa root development and nodule nitrogen fixation efficiency, leading to a double limitation of phosphorus and nitrogen nutrition, and a significant reduction in forage yield and quality [14–16]. These four abiotic stresses have become the core bottlenecks of alfalfa industry development, and it is urgent to identify key regulatory genes responsive to multiple stresses for alfalfa genetic improvement.

Transcription factors are core regulators of plant stress signal transduction, which coordinate the expression of downstream stress-responsive genes, and are ideal targets for crop multi-stress resistance breeding [17]. The B3 superfamily is a plant-specific transcription factor family, defined by the presence of at least one conserved B3 DNA-binding domain [18, 19]. Based on domain architecture and functional divergence, the B3 superfamily is classified into four subfamilies, namely ARF (AUXIN RESPONSE FACTOR), LAV (LEAFY COTYLEDON2 (LEC2)-ABSCISIC ACID INSENSITIVE3 (ABI3)-VAL), RAV (RELATED TO ABI3 and VP1), and REM (REPRODUCTIVE MERISTEM) [18].

*B3* transcription factors play critical roles in regulating plant growth, development and stress responses. The ARF subfamily, the second largest subfamily of B3 superfamily, mainly mediates auxin signal transduction by binding to auxin-responsive elements (AuxREs) in target gene promoters, and is a key node linking hormone signaling and stress adaptation [20]. Numerous studies have reported that ARF subfamily members regulate abiotic stress responses in various plants. For example, silencing of *SlARF2* in *Solanum lycopersicum* enhanced antioxidant enzyme activity and improved tolerance to drought and salt-alkali stress [21]. In *Cucumis sativus*, CsARF5 was identified as a positive regulator of cold stress responses [22]. In *Arabidopsis thaliana* and cereal crops, *ARF10* and *ARF16* have been characterized as key transcription factors that mediate the phosphate starvation response and drought stress adaptation, respectively [23, 24]. The LAV subfamily members are mainly involved in seed development and growth phase transition [25–29], and also act as key regulators in abiotic stress response pathways. For instance, heterologous overexpression of *ScABI3* enhanced salt resistance in transgenic alfalfa by activating ABA signaling and salt-tolerance pathways [30]. Overexpression of flax *LuABI3-1* or *LuABI3-2* also significantly boosted tolerance to salt and mannitol-induced drought stress in *Arabidopsis thaliana* [29]. Some RAV subfamily members harbor both AP2 and B3 DNA-binding domains, enabling specific binding to bipartite cis-elements in target gene promoters [31, 32], with functional studies verifying their regulatory roles in stress responses. For example, heterologous overexpression of cotton *GhRAV1* in *Arabidopsis thaliana* increased hypersensitivity to drought and salt stress, and negatively regulated plant stress tolerance [33], while rice OsRAV9 and OsRAV2 function as negative and positive regulators of drought tolerance, respectively [34, 35]. The REM subfamily is the largest but least functionally characterized subfamily of the B3 superfamily, with most reported members focusing on the regulation of plant reproductive development [36, 37]. Recent study has also revealed that cotton *GhREM1* and *GhREM5.4* play potential regulatory roles under low and high temperature stress [38]. Although the functions of the B3 superfamily have been well characterized in model plants and several crop species, including *Arabidopsis thaliana* [18], *Medicago truncatula* [39], *Glycine max* [40], *Capsicum annuum* [41], *Cucumis sativus* [42], and *Sorghum bicolor* [43], systematic genome-wide identification and functional investigation of this family in alfalfa are still lacking. In particular, the expression patterns and regulatory mechanisms of *MsB3* genes under low-phosphorus, salt, drought, and cold stresses have not been reported to date.

In this study, we performed a genome-wide systematic identification of B3 superfamily members in alfalfa, and performed comprehensive analyses of their phylogenetic relationships, gene structures, conserved domains, and collinearity. We further characterized their expression profiles in different tissues and under four abiotic stresses. Our findings will not only provide insights into the functional divergence of *MsB3* transcription factors, but also offer candidate genes for genetic improvement of multi-stress tolerance in alfalfa.

## Materials and methods

### Identification and physicochemical characterization of *MsB3* genes in alfalfa

The genome of alfalfa cultivar Zhongmu No.1 was obtained from the Alfalfa Genome Project (https://figshare.com/articles/dataset/Medicago_sativa_genome_and_annotation_files/12623960) [5]. Genomes of *Arabidopsis thaliana*, *Medicago truncatula*, and *Glycine max* were retrieved from TAIR (https://www.arabidopsis.org), Medica Go Genome (http://www.medicagogenome.org), and SoyBase (https://www.soybase.org), respectively [44–46].

Using the B3 protein of *Arabidopsis thaliana* as reference sequences, candidate B3 superfamily members in alfalfa were initially identified by BLASTP alignment against the alfalfa proteome with an E-value threshold set to 1e^-5^. The Hidden Markov Model (HMM) profile of the B3 domain (PF02362) was downloaded from the Pfam database and used to remove redundant sequences [47]. All candidate proteins were further submitted to the NCBI Conserved Domain Database (CDD) to verify the presence of the B3 conserved domain. Physicochemical properties of MsB3 proteins were analyzed using the ProtParam tool on the ExPASy server. Subcellular localization was predicted using two online tools: Cell-PLoc 2.0 (http://www.csbio.sjtu.edu.cn/bioinf/Cell-PLoc-2) and WoLF PSORT (https://wolfpsort.hgc.jp).

### Phylogenetic and structural analyses of *MsB3* genes in alfalfa

Multiple sequence alignment of the full-length B3 protein sequences from *Arabidopsis thaliana* and *Medicago sativa* was performed using the MUSCLE algorithm with default parameters. A neighbor-joining (NJ) phylogenetic tree was constructed using MEGA 11.0 software, with the reliability of the tree topology evaluated via bootstrap analysis with 1,000 replicates. Conserved motifs of MsB3 proteins were predicted using the MEME tool, with the parameter set to detect 10 motifs. Gene structural information was extracted from the alfalfa genome GFF annotation file. Conserved motifs and gene structures were visualized using TBtools v2.119 [48].

### Chromosomal localization, collinearity and cis-element analysis

Chromosomal location information of *MsB3* genes was extracted from the GFF annotation file of the alfalfa Zhongmu No.1 reference genome, and visualized using TBtools (v2.119). Tandem duplication events were defined as two or more genes located within a 200 kb region on the same chromosome. Collinearity analysis was performed using MCScanX [49], including intraspecific collinearity within the alfalfa genome, and interspecific collinearity between *Medicago sativa* and other species (*Arabidopsis thaliana*, *Medicago truncatula*, and *Glycine max*).

### Plant materials and stress treatments

Seeds of alfalfa (*Medicago sativa* L.) cultivar Zhongmu No. 1, an independently bred cultivar provided by the Institute of Animal Science, Chinese Academy of Agricultural Sciences, were germinated and grown in a climate-controlled growth chamber with a 16 h light / 8 h dark photoperiod, a day/night temperature regime of 24 ℃/22 ℃, and a relative humidity of 70–80%. After 7 days of germination, uniform seedlings were transferred to 1/2 Hoagland’s nutrient solution for hydroponic culture for 4 weeks.

Three individual abiotic stress treatments were imposed: cold stress (4 °C), salt stress (250 mM NaCl), and drought stress (simulated by 400 mM mannitol). Samples were collected at 0, 2, 4, 6, 24, and 48 h for cold stress, and at 0, 1, 3, 6, 12, and 24 h for salt and drought stresses. At each time point, leaves from three biological replicates were collected, immediately snap-frozen in liquid nitrogen, and stored at − 80 °C until further use.

For low-phosphorus treatment, stem nodal cuttings of alfalfa were first rooted in vermiculite. Uniform rooted seedlings were pre-cultured in full nutrient solution containing 500 µmol/L Pi for 3 weeks. For transcriptome sequencing, the seedlings were then randomly divided into two groups and grown for another 3 weeks in either sufficient-phosphorus (500 µmol/L Pi, +P) or low-phosphorus (0 µmol/L Pi, -P) nutrient solution. Root tissues from three biological replicates per treatment were sampled. For the low‑phosphorus time‑course qRT‑PCR analysis, seedlings pre‑cultured under the same conditions were transferred to -P nutrient solution, and root samples were collected at 0, 2, 6, 12, 24 and 48 h after transfer, with five biological replicates at each time point. All harvested root samples were immediately snap‑frozen in liquid nitrogen and stored at − 80 °C until further use.

### Transcriptome analysis of *MsB3* genes in alfalfa

#### Expression pattern analysis under salt, drought and cold stresses

Publicly available RNA-seq data were downloaded from the National Center for Biotechnology Information (NCBI) Sequence Read Archive (SRA) database, including datasets from six alfalfa tissues (SRP055547) and datasets from alfalfa seedlings under cold, drought, and salt stresses (SRR7091780–SRR7091794, SRR7160313–SRR7160357) [50, 51]. Clean reads were aligned to the alfalfa reference genome (cultivar Zhongmu No. 1), and transcript abundance was quantified using FeatureCounts with fragments per kilobase of transcript per million mapped reads (FPKM) normalization. Expression profiles were visualized using TBtools v2.119.

#### Expression pattern analysis under + P and -P conditions

Total RNA was extracted from alfalfa root samples under + P and -P treatments, with three biological replicates per treatment, and used for cDNA library construction. The libraries were paired-end sequenced on the Illumina NovaSeq 6000 platform. Clean reads were aligned to the alfalfa reference genome (cultivar Zhongmu No. 1) using HISAT2 (v2.0.5). Gene expression levels were quantified with FeatureCounts (v1.5.0-p3) and normalized to FPKM values.

### RNA Extraction and qRT-PCR analysis

Total RNA was extracted from all samples using the Eastep Super Total RNA Extraction Kit (Promega). First-strand cDNA was synthesized from 1 µg of total RNA using the PrimeScript II 1st Strand cDNA Synthesis Kit (Takara). Quantitative real-time PCR (qRT-PCR) was performed on the CFX96 Real-Time PCR System with SYBR Green qPCR Premix (Vazyme). Each reaction was performed in three replicates. Relative expression levels were normalized to the *MsActin* reference gene and calculated using the 2^−ΔΔCt^ method. The primers were listed in Table S1.

### Statistical analysis

All data were presented as mean ± standard deviations (SDs). Graphs were generated using GraphPad Prism 9.5 software and TBtools v2.119. Statistical significance was determined by two-tailed Student’s t-test, with *P* < 0.05 considered statistically significant.

## Results

### Identification and physicochemical property analysis of *MsB3* genes in alfalfa

A total of 146 *MsB3* genes were identified in the genome of alfalfa cultivar Zhongmu No. 1 via BLASTP homology search against known B3 proteins of *Arabidopsis thaliana*, combined with hidden Markov model (HMM) verification and conserved domain confirmation. These genes comprised 89 REM, 7 RAV, 8 LAV, and 42 ARF members. The coding sequences (CDS) and corresponding protein sequences of these genes are listed in Table S2. The 146 *MsB3* genes were renamed according to their chromosomal locations and family classifications, referring to previous studies [42, 43].

**Fig. 1:**
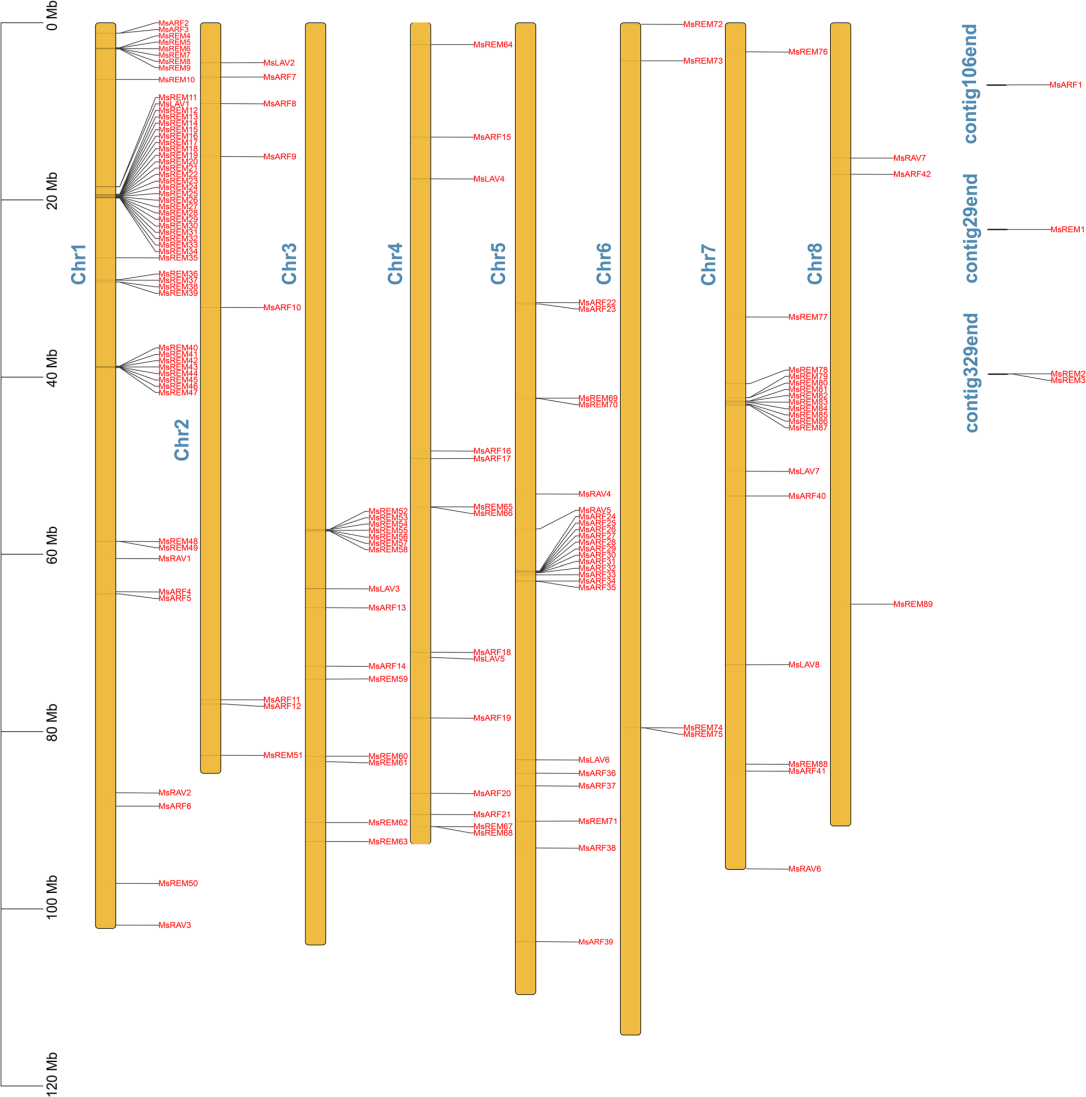
Chromosome distribution of the *MsB3* genes in alfalfa. The chromosomes of alfalfa cultivar Zhongmu No. 1 are represented by yellow vertical bars, with the corresponding chromosome numbers marked in blue font. A scale bar indicating the physical length of chromosomes is shown on the left

As shown in Fig. 1, the *MsB3* genes were distributed across all eight chromosomes, with four genes unmapped to the conventional chromosomes. Chromosome 1 (chr1) harbored the largest number of *MsB3* genes (56 genes), accounting for 55% of all REM members. In contrast, chromosome 8 (chr8) contained only three genes, *MsRAV7*, *MsARF42*, and *MsREM89*.

The physicochemical properties and predicted subcellular localizations of the MsB3 proteins showed significant differences. As shown in Table S3, *MsREM76* encodes the shortest protein among the B3 superfamily members, with only 65 amino acids (aa) and a molecular weight of 7.56 kDa, whereas *MsLAV3* encodes the longest protein, consisting of 1581 aa and a molecular weight of 176.05 kDa. Notably, the amino acid length between these two proteins differs by approximately 24-fold. Most MsB3 proteins were alkaline, with 85 members having an isoelectric point (pI) greater than 7.5, while 49 were acidic (pI < 6.5) and 12 were neutral (6.5 ≤ pI ≤ 7.5). The instability index ranged from 18.3 to 71.71, and 63 members had an instability index lower than 40, being classified as biochemically stable. For the grand average of hydropathicity (GRAVY), only one protein showed a positive GRAVY value, indicating that the vast majority of MsB3 proteins are hydrophilic. Moreover, subcellular localization prediction indicated that 83 members were localized in the nucleus, the primary subcellular compartment. 29 and 23 members were predicted to localize in the cytoplasm and chloroplast, respectively, while the remaining proteins were distributed in compartments including mitochondria, vacuoles and others.

### Phylogenetic analysis of B3 superfamily members in alfalfa

**Fig. 2:**
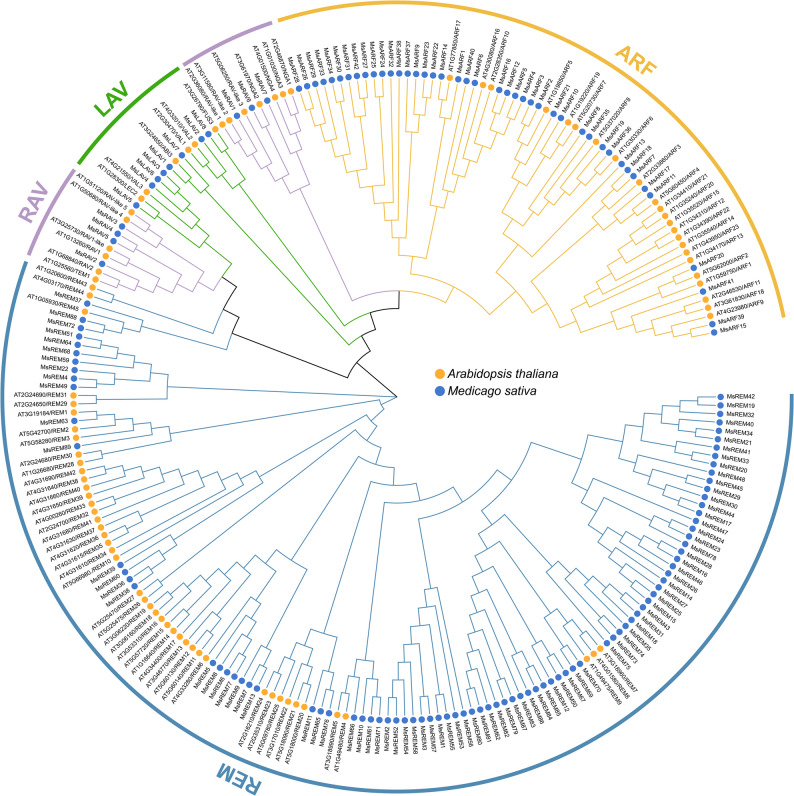
Phylogenetic tree of the MsB3 proteins. The four subfamilies of the B3 superfamily are highlighted with distinct colors. Yellow and blue symbols represent *Arabidopsis thaliana* and *Medicago sativa proteins*, respectively

To clarify the classification and evolutionary relationships of the B3 superfamily members, a neighbor-joining phylogenetic tree was constructed using B3 protein sequences from *Medicago sativa* and *Arabidopsis thaliana*. As shown in Fig. 2, the phylogenetic tree classified the alfalfa B3 superfamily into four distinct subfamilies, which was consistent with previous studies [42, 43]. Among these, the REM subfamily was the largest with 89 members, followed by the ARF (42 members), LAV (8 members), and RAV (7 members) subfamilies.

### Motifs and gene structures analysis of B3 superfamily members in alfalfa

**Fig. 3:**
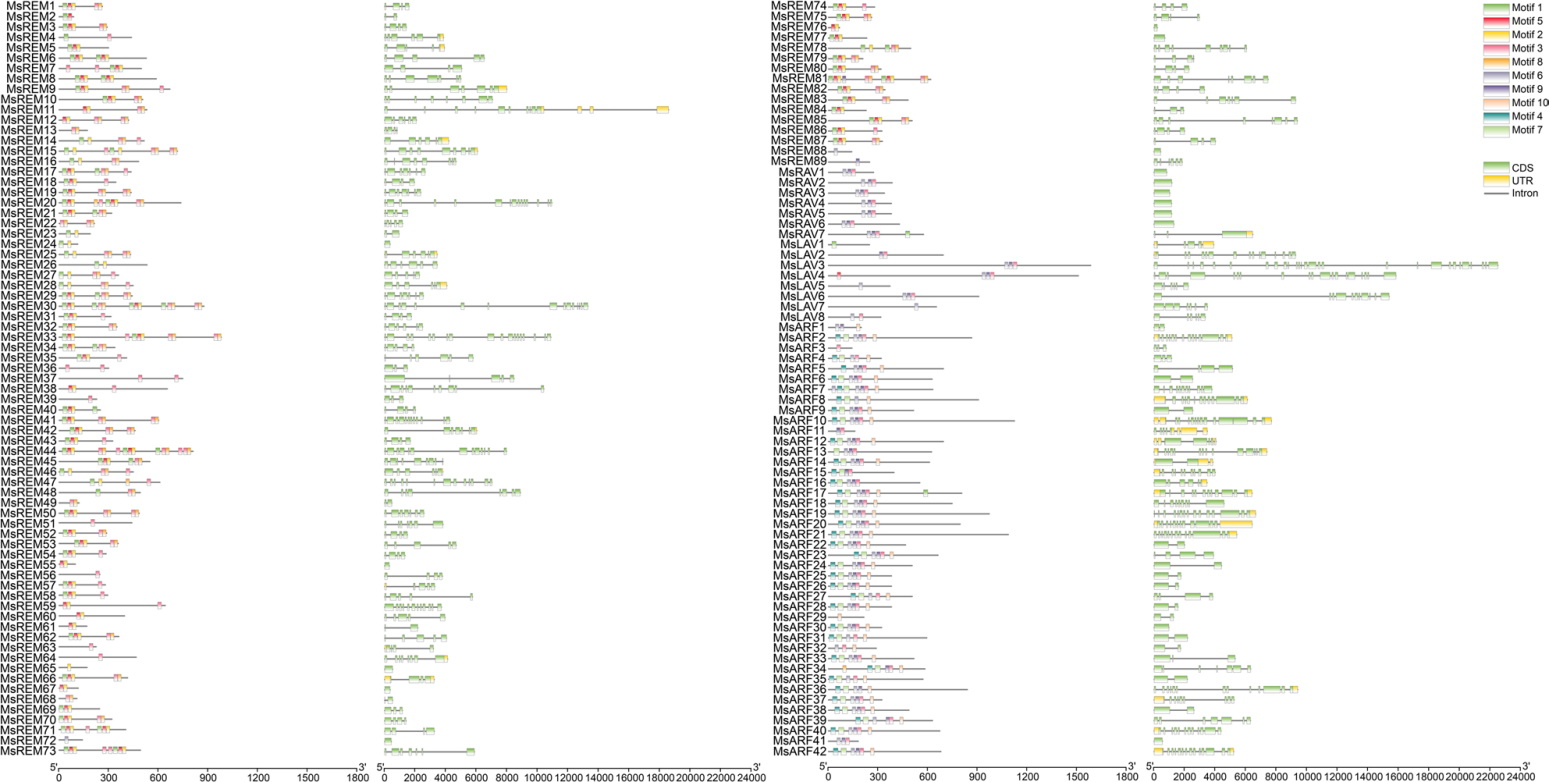
Gene structure and motifs of B3 superfamily members in alfalfa

To characterize the structural features of the alfalfa B3 superfamily, 146 MsB3 protein sequences were analyzed using the MEME suite with the parameter set to identify 10 conserved motifs (Fig. 3). The results showed that most MsREM proteins contained Motif 1, Motif 2, and Motif 5, with a conserved motif arrangement pattern. Members of the RAV and LAV subfamilies contained relatively fewer motifs. Except for MsRAV7, which also harbored Motif 1, all other RAV subfamily members contained only Motif 3, Motif 6, and Motif 9 in a consistent order, indicating that these three motifs were highly conserved in the RAV subfamily. Additionally, Motif 8 and Motif 10 were specifically present in MsARF proteins.

Gene structure analysis revealed that genes in the REM subfamily exhibited substantial variation in exon number. Eight *MsREM* members contained only one exon, whereas *MsREM33* contained 18 exons. In the RAV subfamily, all members contained a single exon, except *MsRAV7*, which possessed three exons. Notably, *MsLAV3* had the highest exon number (28), which may increase the diversity of alternative splicing and enhance functional flexibility in alfalfa. Most ARF subfamily genes had relatively dispersed CDS regions, with 14 members containing more than 10 exons.

### Gene duplication events and collinearity analysis of *MsB3* genes in alfalfa

**Fig. 4:**
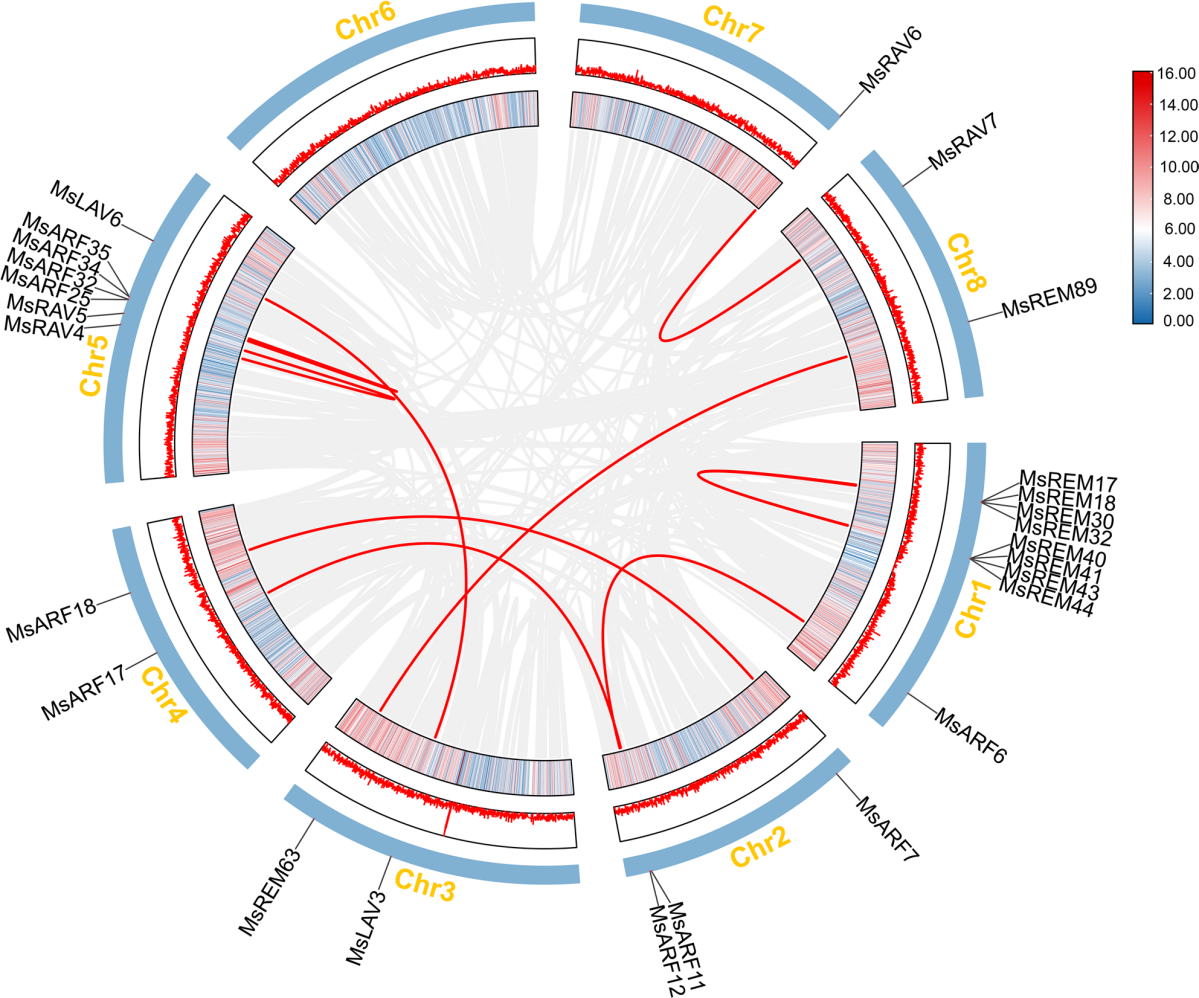
Collinearity analysis of *MsB3* genes in alfalfa. Gray lines connect the collinear pairs across the alfalfa genome, whereas red curves highlight the segmental duplication events within the B3 superfamily. The innermost layer illustrates the gene density across chromosomes, while the middle layer represents the gene abundance distribution. A unified scale on the right side is provided to quantify both gene density and abundance

Gene duplication events play critical roles in promoting the expansion and functional evolution of plant gene families [48]. As shown in Fig. 1 and Table S4, a total of 18 tandem duplication events were present in the alfalfa B3 superfamily, involving 79 genes, among which 65 were *MsREM* genes and 14 were *MsARF* genes. The distribution of tandem duplications varied greatly among chromosomes. Chromosome 1 (chr1) contained the largest number of tandem duplication events, amounting to 7 events (e.g., *MsARF2*/*MsARF3*), and tandem duplication was observed on chr 2 and chr8. In addition, as displayed in Fig. 4 and Table S5, 13 segmental duplication events were identified in the B3 superfamily, involving 26 genes (e.g., *MsARF6* on chr1 and *MsARF12* on chr2). The distinct distribution patterns of tandem and segmental duplications imply that these two duplication types may have contributed differently to the evolutionary expansion of the alfalfa B3 superfamily.

**Fig. 5:**
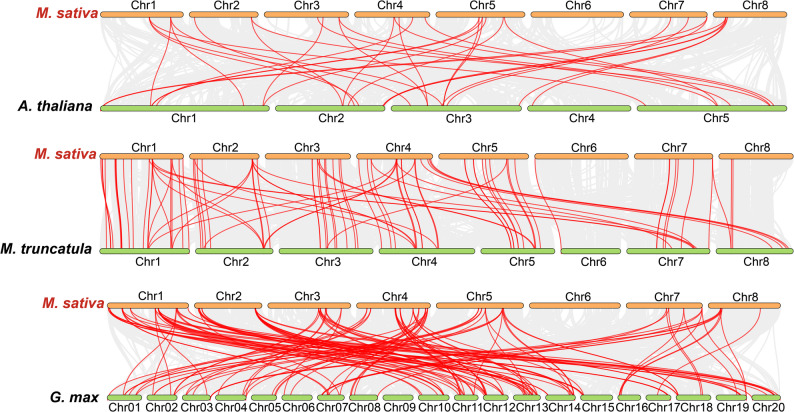
Collinearity analysis of *MsB3* genes among *Medicago sativa* (*M*. *sativa*), *Arabidopsis thaliana* (*A*. *thaliana*), *Medicago truncatula* (*M*. *truncatula*) and *Glycine max* (*G*. *max*). Gray lines represent collinear pairs between the compared genomes, whereas red lines highlight duplicated gene pairs

To further reveal the evolutionary relationships of the B3 superfamily between *Medicago sativa* and other species, interspecific collinearity analyses were performed with *Arabidopsis thaliana*, *Medicago truncatula* and *Glycine max* (Fig. 5). The results indicated that 21 *MsB3* genes exhibited collinear relationships with all three species, and 20 genes were collinear with both *Medicago truncatula* and *Glycine max*. In total, 34, 98 and 128 collinear gene pairs were identified between *Medicago sativa* and *Arabidopsis thaliana*, *Medicago truncatula* and *Glycine max*, respectively, indicating evolutionary conservation of the B3 superfamily among legume species.

### Analysis of cis-acting elements in B3 superfamily members of alfalfa

To elucidate the potential transcriptional regulatory functions of the B3 superfamily in alfalfa, the cis-acting elements within the 2000 bp promoter regions upstream of the *MsB3* genes were predicted and analyzed using the PlantCARE online tool. The results revealed that the identified cis-acting elements could be classified into four functional categories, namely light-responsive, hormone-responsive, growth and development regulatory, and stress-responsive elements. Notably, light-responsive elements were detected in the promoter regions of all B3 superfamily members (Table S6).

**Fig. 6:**
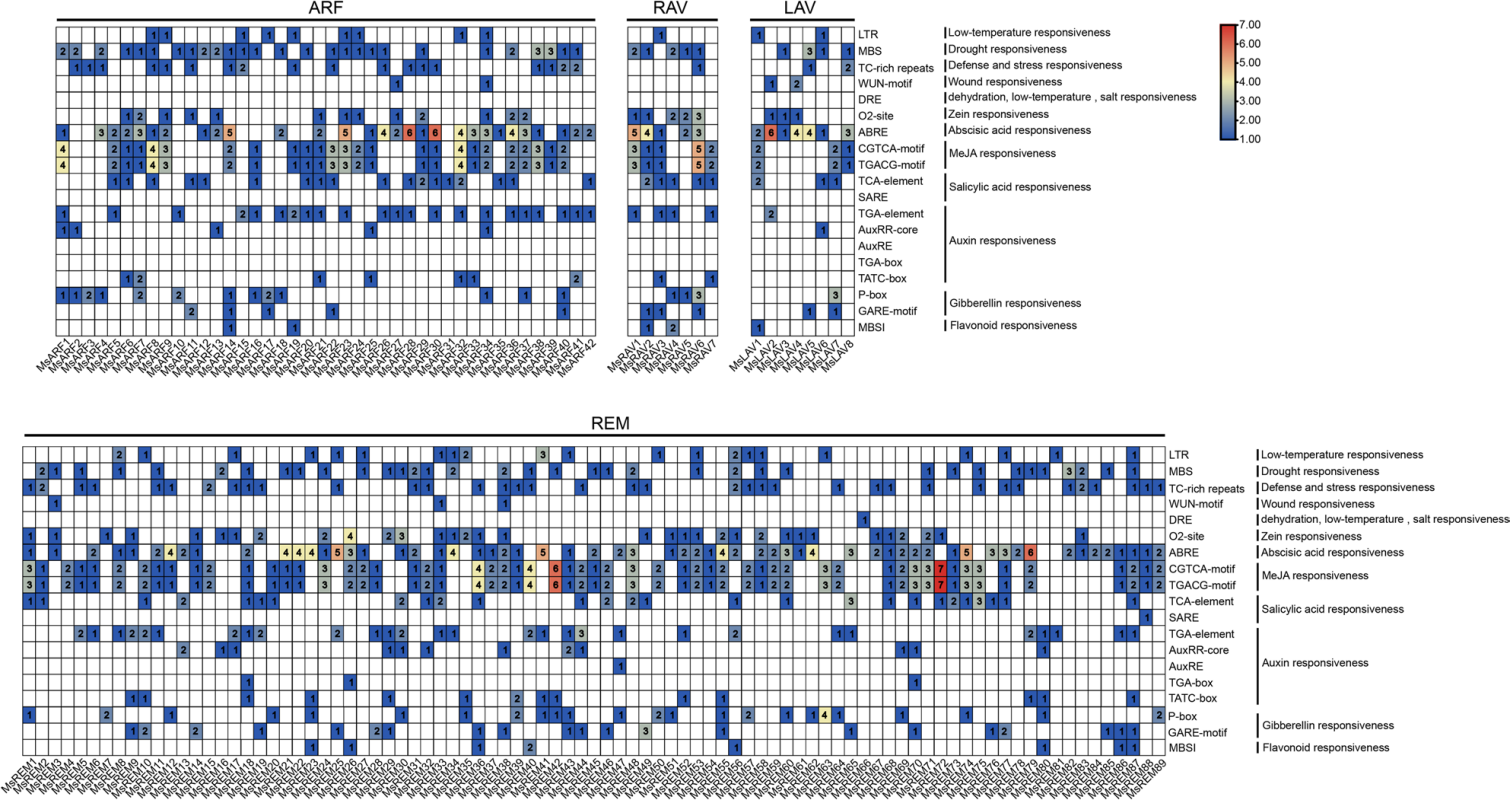
Analysis of cis‑acting elements in the promoter regions of *MsB3* genes. According to the phylogenetic tree, *MsB3* genes are assigned into four subfamilies, namely ARF, RAV, LAV and REM. The scale bar on the right indicates the count of cis‑acting elements present in each promoter

As shown in Fig. 6, approximately 69% of *MsB3* gene promoters contained the abscisic acid-responsive element (ABRE), which was the most abundant element type. In the ARF subfamily, auxin-responsive elements were present in the promoters of 28 members, while the drought-responsive element MBS was distributed in 27 members. Moreover, the promoter of *MsARF30* contained one type of cis-acting element, which was a salicylic acid-responsive element. In the RAV subfamily, 5 members harbored ABRE, MeJA-responsive elements, and salicylic acid-responsive elements in their promoters, respectively. No stress-responsive elements were detected in the promoters of *MsRAV7* and *MsLAV7*, whose promoter regions were only enriched with hormone-responsive elements. In the REM subfamily, *MsREM66* was the only gene containing the dehydration, low-temperature, and drought-responsive element (DRE) in its promoter. In contrast, *MsREM72* lacked any stress-responsive elements but contained 14 MEJA-responsive elements, representing the highest number of this element among the entire B3 superfamily.

### Expression patterns of *MsB3* genes in different tissues of alfalfa

**Fig. 7:**
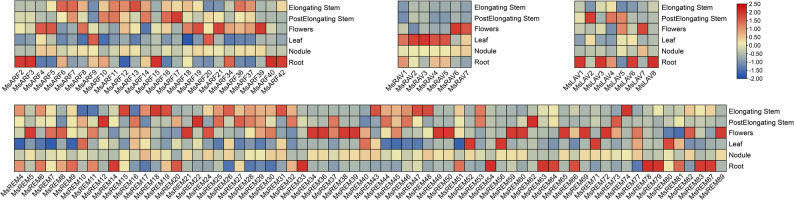
Expression profiles of *MsB3* genes in different alfalfa tissues. The color bar on the right of the heatmap represents the relative expression levels of the genes, with a blue-to-red color gradient corresponding to a gradual increase in expression levels

To clarify the expression patterns of *MsB3* genes across different tissues of alfalfa, expression levels were retrieved from the public NCBI database for six tissues: elongated stems, post-elongated stems, flowers, leaves, nodules and roots (Table S7). The results revealed that 109 *MsB3* genes were expressed in at least one of the six tissues. Among them, three genes exhibited tissue-specific expression patterns including *MsREM54*, *MsREM62* and *MsREM78*, while 73 genes were expressed in all six tissues (Fig. 7). Furthermore, some *MsB3* genes showed significant differences in expression abundance among six tissues. For instance, *MsARF19* exhibited the highest expression levels in flowers, elongated stems, and nodules, with a relative expression level of only 30.46 in roots. *MsRAV2* was expressed at a relative level of 178.11 in leaves but only 3.89 in flowers. *MsARF15* showed the highest expression level among all *MsB3* genes in roots.

### Expression patterns of *MsB3* genes in alfalfa under different abiotic stresses

**Fig. 8:**
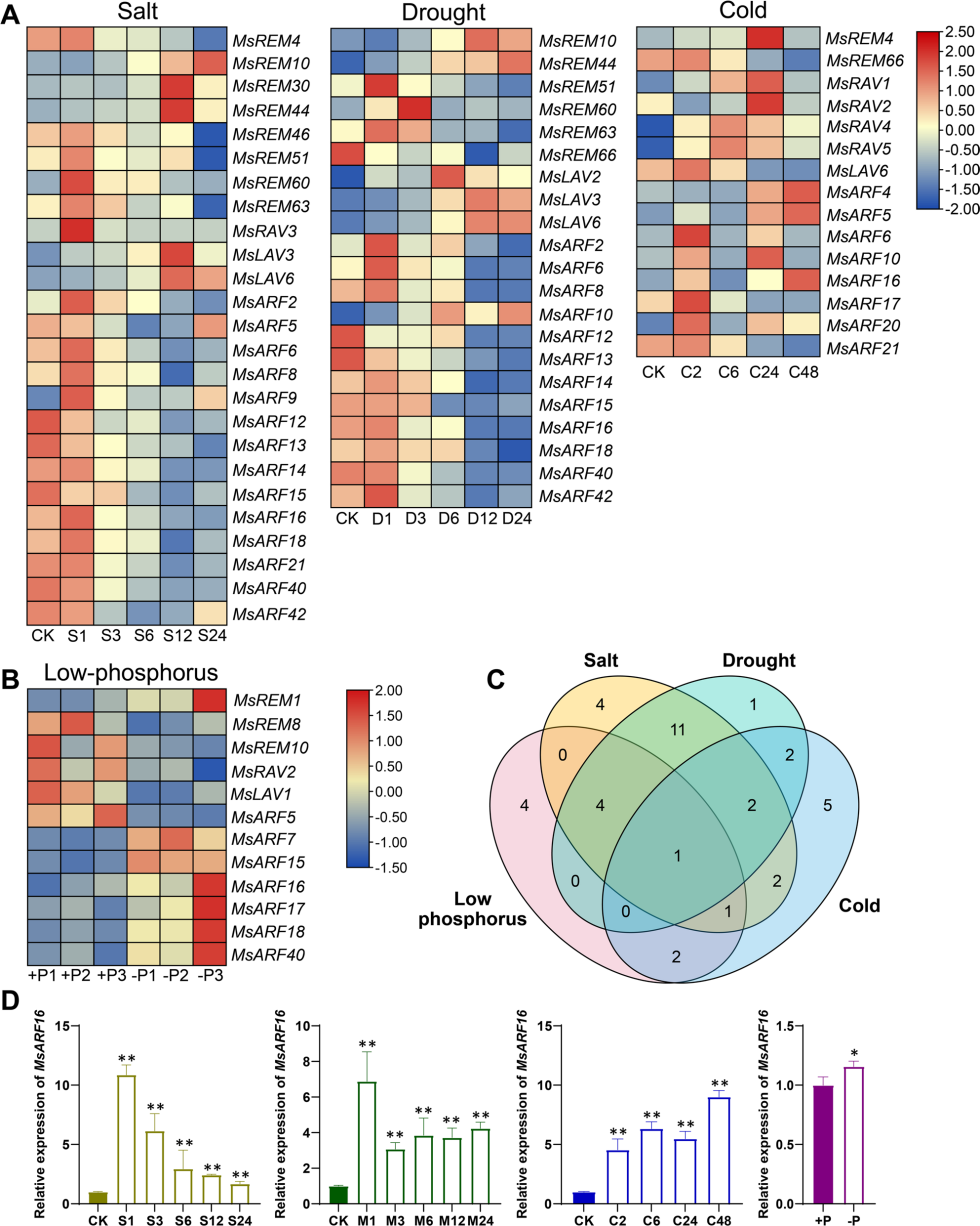
Expression profiles of *MsB3* genes in alfalfa under abiotic stresses. **A** Heatmaps of *MsB3* gene expression under salt, drought, and cold stresses. **B** Heatmap of *MsB3* gene expression under sufficient-phosphorus (+ P) and low-phosphorus(-P) conditions. **C** Venn diagram of overlapping differentially expressed *MsB3* genes across four stresses. **D** qRT-PCR analysis of *MsARF16* expression under four abiotic stresses. Significance was evaluated using a two-tailed Student’s t-test, with *P < 0.05 and **P < 0.01

To characterize the stress‑responsive expression patterns of the B3 superfamily in alfalfa, transcriptome data from plants exposed to salt, drought, cold, and low-phosphorus (-P) stresses were analyzed (Table S8). The results showed that the number of stress‑responsive *MsB3* genes differed significantly across treatments. As illustrated in Fig. 8A and B, salt stress induced the largest number of responsive genes (25 genes), whereas -P stress induced the fewest (12 genes). A total of 14 *MsB3* genes were specifically responsive to only one stress (Fig. 8A-C). For example, *MsLAV2* was uniquely induced by drought stress and showed significantly upregulated expression. A total of 15 genes responded to exactly two stresses, among which genes co‑responsive to salt and drought stresses were the most numerous (11 genes). Notably, no *MsB3* genes were specifically co‑responsive to -P and salt stresses, or to -P and drought stresses. Moreover, seven genes responded to three stresses: *MsARF18*, *MsREM10*, *MsARF15*, and *MsARF40* were co‑responsive to -P, salt and drought stresses; *MsARF5* was co‑responsive to -P, drought and cold stresses; and *MsARF6* and *MsLAV6* were co‑responsive to salt, drought and cold stresses. Only *MsARF16* responded to all four stresses, with upregulated expression under -P and cold stresses but downregulated expression under salt and drought stresses. These results indicated that *MsARF16* may act as a candidate gene in the alfalfa stress-response regulatory network, and its regulatory mechanism and downstream pathways deserve further in-depth investigation. Therefore, *MsARF16* was selected for qRT-PCR to verify the reliability of the transcriptome-derived expression profiles (Fig. 8D). The qRT-PCR results were consistent with the transcriptome trends. Under salt and drought stresses, the relative expression of *MsARF16* increased first and then decreased with prolonged treatment, while under cold stress, it peaked at 48 h. Under low‑phosphorus stress, a modest increase was detected at 3 weeks, and a 48‑h time‑course analysis showed an overall upward trend (Fig. S1), indicating that *MsARF16* is responsive to low‑phosphorus stress. These findings confirmed the reliability of our transcriptome dataset.

## Discussion

Abiotic stresses, including salt, drought, cold and phosphorus deficiency, are the major limits to yield and quality stability in alfalfa [8, 9]. The plant-specific B3 transcription factor superfamily plays important roles in plant growth, development and abiotic stress responses [18], but its systematic characterization and stress-responsive functions in alfalfa remain poor, severely limiting its use in alfalfa stress tolerance breeding.

In this study, we identified 146 B3 superfamily members in the genome of alfalfa cultivar Zhongmu No.1. These members were classified into ARF, RAV, LAV and REM subfamilies, consistent with previous findings [42, 43]. The number of B3 superfamily members in alfalfa is significantly higher than that in *Arabidopsis thaliana*, which is closely related to the autotetraploid genome characteristics and genome duplication events of alfalfa [5, 18]. Among the four subfamilies, the REM subfamily is the largest with 89 members, accounting for more than 60% of the total *MsB3* genes, which is consistent with the dominant expansion of the REM subfamily in most angiosperms [42, 43]. Analysis of gene duplication events showed that these events were the major driving force for the expansion of the B3 superfamily in alfalfa, especially for the REM subfamily. A total of 65 *REM* genes were involved in tandem duplication events, and 10 in segmental duplication events. This differential expansion pattern suggests that tandem duplication and segmental duplication collectively shaped the evolutionary scale and functional divergence of the B3 superfamily in alfalfa. Interspecific collinearity analysis showed that *MsB3* genes had more collinear gene pairs with legume species *Medicago. truncatula* (98 pairs) and *Glycine max* (128 pairs) than with *Arabidopsis. thaliana* (34 pairs), which not only reflects the high evolutionary conservation of the B3 superfamily among legumes, but also provides functional inference of *MsB3* genes based on experimentally validated functions of their homologs in model legumes.

Conserved motif and gene structure analyses further supported the phylogenetic classification of the B3 superfamily in alfalfa. Members within the same subfamily had conserved motif composition and exon-intron structure, which is consistent with the structural features of the B3 superfamily in other plant species [42, 43], suggesting that the core functions of each subfamily are relatively conserved during evolution. For example, all RAV subfamily members except *MsRAV7* contained a single exon and a consistent arrangement of subfamily-specific motifs, which ensures their stable recognition and binding to target cis-elements to exert transcriptional regulatory functions [32]. Meanwhile, extensive variations in gene architecture are observed across different subfamilies, especially in the REM subfamily with the number of exons ranging from 1 to 18. Such variation in exon-intron structure may contribute to the functional differentiation of REM members, supporting their involvement in diverse biological processes, including the conserved regulation of reproductive meristem development [36, 37] and responses to abiotic stresses [38].

Cis-acting elements in the promoter region are key regulators of the spatiotemporal expression and stress-responsive patterns of genes [52]. Hormone-responsive and stress-responsive elements are widely distributed in promoters o regions of *MsB3* genes. Notably, the ABA-responsive element (ABRE) was present in 69% of *MsB3* gene promoters, suggesting this family may mediate abiotic stress responses through the ABA signaling pathway [53]. In the ARF subfamily, 28 members harbored auxin-responsive elements and 27 contained drought-responsive MBS elements in their promoters, with the coexistence of these elements suggesting such *MsARF* genes integrate auxin and stress signaling to regulate adaptive growth of alfalfa under adverse conditions. We further identified several genes with unique cis-element distribution profiles in their promoter regions. For example, Specifically, *MsRAV7* and *MsLAV7* only contained hormone-responsive elements in their promoters, which is consistent with their high expression in flowers and non-response to the four abiotic stresses, suggesting their potential roles in hormone-regulated floral development. Methyl jasmonate (MeJA) is an important phytohormone regulating plant reproductive development, secondary metabolism and biotic stress responses [54]. *MsREM72* contained no stress-responsive elements but 14 MeJA-responsive elements, suggesting that it may be specifically involved in MeJA-mediated biological processes in alfalfa rather than abiotic stress responses.

Tissue expression profile analysis showed that the potential biological functions of *MsB3* genes in alfalfa growth and development. A total of 73 *MsB3* genes were expressed in all six examined tissues, indicating that these members are widely involved in the whole growth and development process of alfalfa. The tissue-specific expression patterns of many *MsB3* genes were consistent with the conserved functions of their subfamily members reported in model plants. For example, in *Arabidopsis thaliana*, ARF7 and ARF19 regulate lateral root initiation and development, with the *arf7 arf19* double mutant showing severe impairment in lateral root formation [55]. MtARF2, MtARF3 and MtARF4 regulate lateral root development and rhizobia symbiotic nodule formation in *Medicago truncatula* [56]. Correspondingly, *MsARF2*, *MsARF3*, *MsARF4* and other *MsARF* members were highly expressed in alfalfa roots and nodules, implying their potential roles in root and nodule development. For the RAV subfamily, overexpression of *AtRAV1* leads to retarded rosette leaf development, smaller leaves and accelerated senescence in *Arabidopsis* [57, 58]. In alfalfa, *MsRAV2*, *MsRAV4* and *MsRAV5*, which are phylogenetically close to *AtRAV1*, exhibited the highest expression levels in alfalfa leaves, suggesting these genes may also participate in the regulation of leaf development. In addition, previous studies have reported that REM subfamily members are mainly involved in plant reproductive development [59, 60]. Tissue expression data showed that most *MsREM* genes were highly expressed in flowers, further suggesting that the REM subfamily also plays vital roles in alfalfa reproductive development.

Salt, drought, cold and low-phosphorus stresses are major abiotic factors that severely restrict alfalfa yield and quality [8, 9]. Systematic analysis of the expression patterns of the alfalfa B3 superfamily was performed under these four stresses. Transcriptome analysis showed that members of the B3 superfamily exhibited both stress-specific and multi-stress co-responsive expression patterns. Salt stress induced the largest number of responsive *MsB3* genes, indicating a more extensive regulatory role of this family in alfalfa salt tolerance. In contrast, low‑phosphorus stress triggered the fewest responsive genes, which may be attributed to the long‑term adaptive nature of low‑phosphorus stress, whereas the other three stresses represent acute stress responses [16]. We further identified a set of key stress-responsive candidate genes through expression profiling. Among them, 14 genes showed single stress-specific response patterns, such as *MsLAV2* which was specifically induced by drought stress, providing valuable targets for the genetic improvement of single stress tolerance in alfalfa. Seven *MsB3* genes responding to three stresses were identified, and only *MsARF16* was responsive to all four stresses, showing up-regulated expression under low-phosphorus and cold stresses and down-regulated expression under salt and drought stresses. Its stress-responsive expression pattern was further validated by qRT-PCR, which showed high consistency with the transcriptome data. Phylogenetic analysis revealed that *MsARF16* is closely related to *Arabidopsis ARF10* and *ARF16*, both of which have been reported to participate in drought response [23]. In rice, a mutant of the *ARF16* ortholog *osarf16* has been shown to reduce sensitivity to low‑phosphorus stress [61]. Additionally, *ZmARF16* and *ZmARF10* were also identified as candidate genes for phosphate deficiency tolerance in maize [24]. In soybean, edited hair roots of *GmARF16* exhibited stronger tolerance to saline‑alkali conditions, with longer primary roots and no leaf chlorosis [62]. These findings from multiple species suggest that *ARF16* orthologs are broadly involved in abiotic stress responses. Taken together, our results indicated that *MsARF16* acts as a candidate gene in the multi‑stress response regulatory network of alfalfa and represents a promising target for the genetic improvement of multi-stress tolerance.

Alfalfa is a globally critical perennial legume forage with prominent economic and ecological values, while increasing abiotic stresses have become a major constraint to its stable yield and quality. The key candidate genes identified in this study, which provide the genetic resources for alfalfa molecular breeding. These findings lay a fundamental basis for further functional verification of B3 superfamily genes and the cultivation of new alfalfa varieties with enhanced multi-stress tolerance and superior agronomic traits.

## Conclusions

In this study, a total of 146 B3 superfamily genes were identified in the alfalfa genome, which were classified into REM, ARF, LAV and RAV subfamilies. The REM subfamily was the largest with 89 members, accounting for more than 60% of the total identified *MsB3* genes. All MsB3 members contained the conserved B3 DNA-binding domain, with subfamily-specific conserved motif distribution and gene structural features observed in the four subfamilies. Gene duplication analysis revealed that both tandem and segmental duplication events drove the expansion of the alfalfa B3 superfamily. Cis-acting element analysis showed that the promoter regions of most *MsB3* genes harbored ABRE elements. Tissue expression profile analysis showed that 109 *MsB3* genes were expressed in at least one of the six alfalfa tissues, among which three genes exhibited tissue-specific expression patterns, while 73 genes were expressed in all six tissues. Expression pattern analysis under abiotic stresses revealed that *MsB3* genes exhibited diverse responsive profiles under salt, drought, cold and low-phosphorus stresses. Specifically, 14 genes were responsive to a single stress, 15 genes responded to two stresses, 7 genes were co-regulated by three stresses, and only *MsARF16* responded to all four stresses. Taken together, this study provides a comprehensive characterization of the B3 superfamily in alfalfa, lays a foundation for further functional exploration of *MsB3* genes, and offers some candidate genes for molecular breeding of alfalfa.

## Supplementary Information

Supplementary Material 1: Fig S1. Expression of *MsARF16* in alfalfa roots during a 48‑h low‑phosphorus treatment. Table S1. Primers of *MsB3* genes used for qRT-PCR. Table S2. CDS and protein sequences of *MsB3* genes identified in this study. Table S3. B3 superfamily genes in alfalfa. Table S4. Tandem duplication events involved in *MsB3* genes in alfalfa. Table S5. Segmental duplication events involved in *MsB3* genes in alfalfa. Table S6. Cis-acting elements in the promoter regions of *MsB3* genes. Table S7. The expression of *MsB3* genes in different tissues. Table S8. Candidate *MsB3* genes under abiotic stresses.

## Data Availability

The datasets generated and analyzed during the current study are available in the NCBI SRA repository under BioProject accession number PRJNA1447123.
